# Supplementary material for: Chromosomal characteristics of salt stress heritable gene expression in the rice genome
Source: BMC Genom Data. 2021 May 27;22:17. doi: 10.1186/s12863-021-00970-7 (PMC8162008; doi:10.1186/s12863-021-00970-7)
Supplement: Supplementary file 1 — Additional file 1. [file 12863_2021_970_MOESM1_ESM.docx]

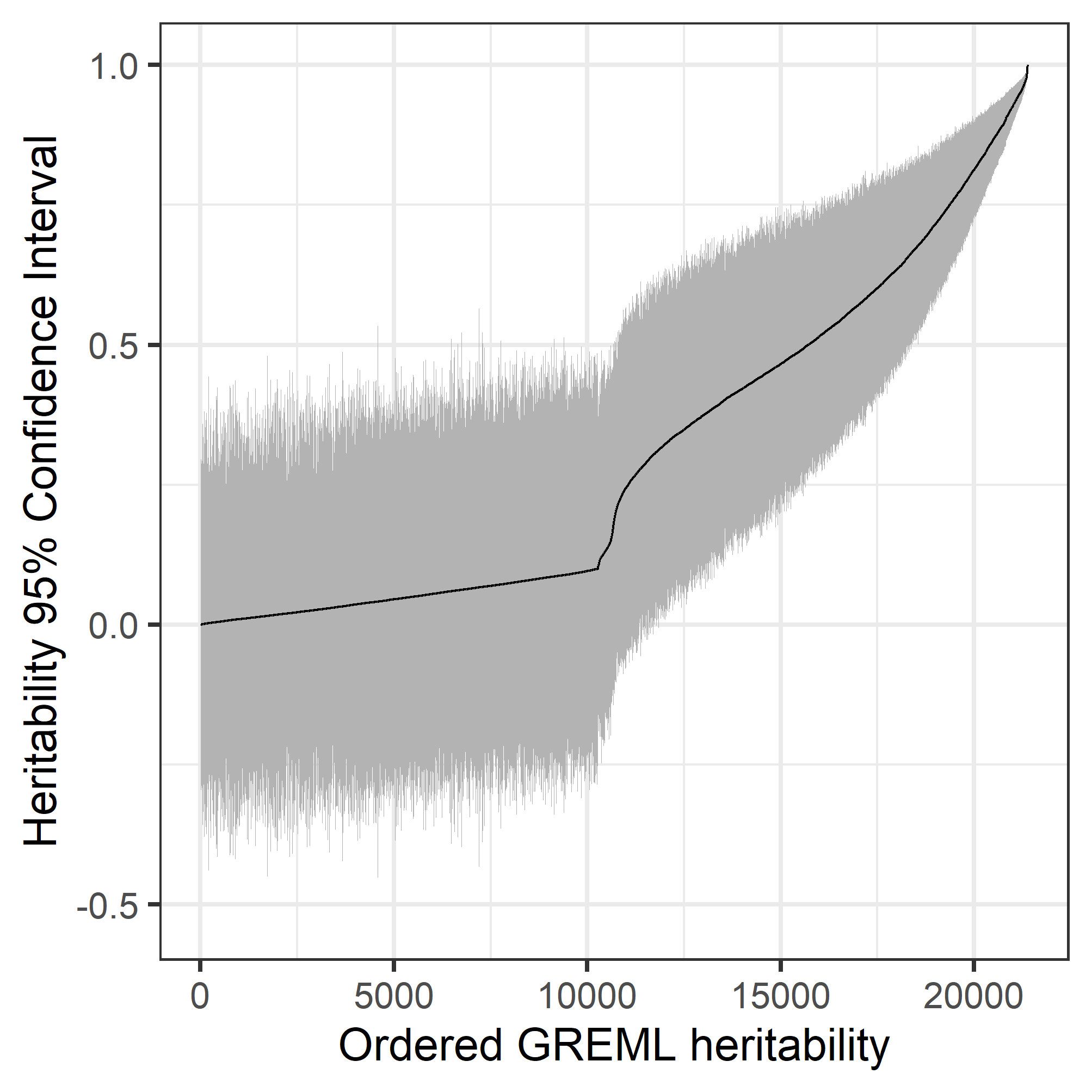


**Figure S1. Single-step GREML Heritability Estimates.** Heritability estimates using the single-step GREML method were ordered and plotted using a black line. The 95% confidence estimates of each heritability estimate, are represented by grey vertical lines.

**
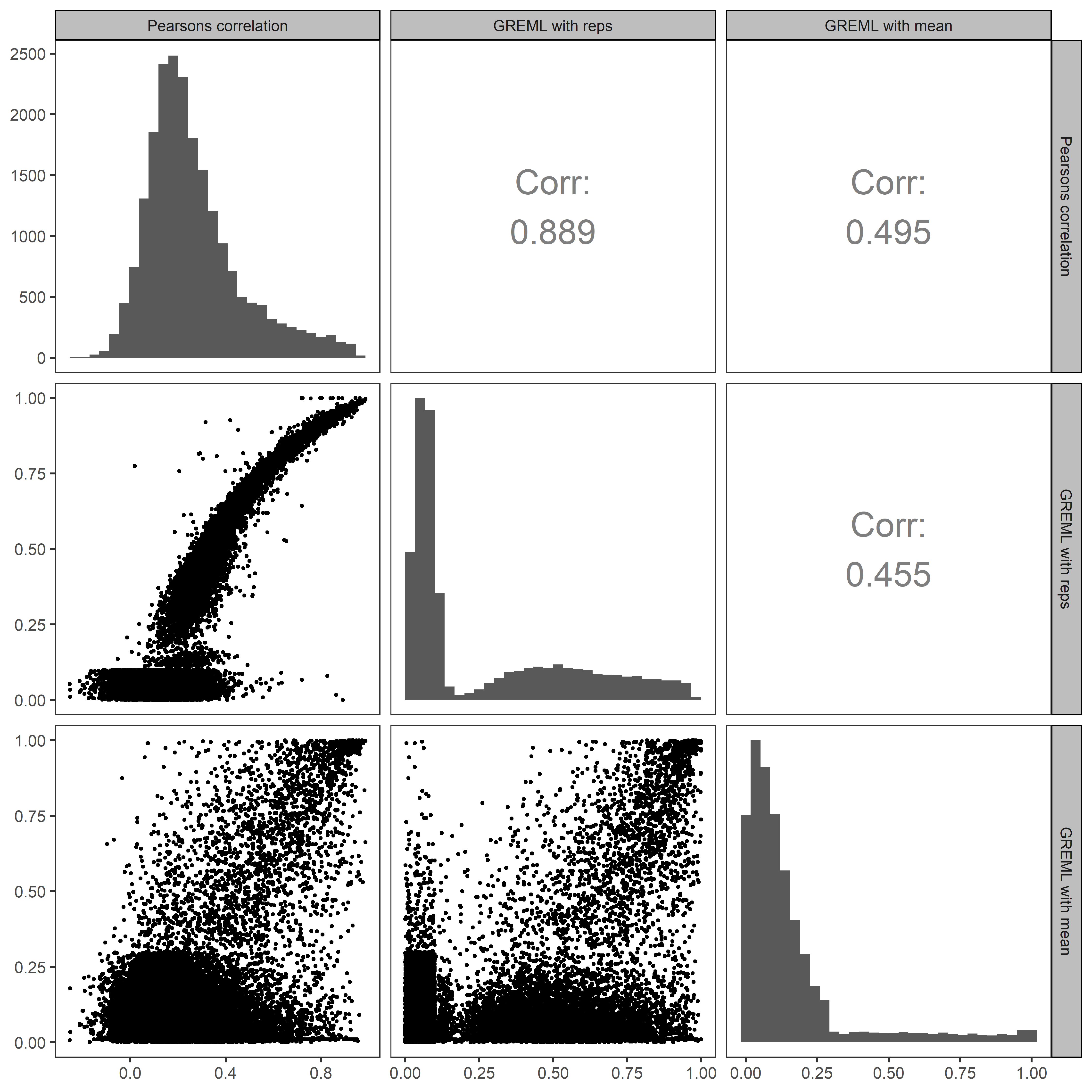
**

**Figure S2. Comparison of Heritability Calculation Methods for Salt-Stress**. Pairwise correlation between repeatability (Pearson), single-step GREML (with replicates), and two-step GREML (using the genotypic mean) for the salt-stress condition. The lower triangle shows correlation scatterplots of the pairwise comparisons, the diagonal provides the density distribution plots for each individual method and the upper right triangle provides the corresponding pairwise correlation values.

**
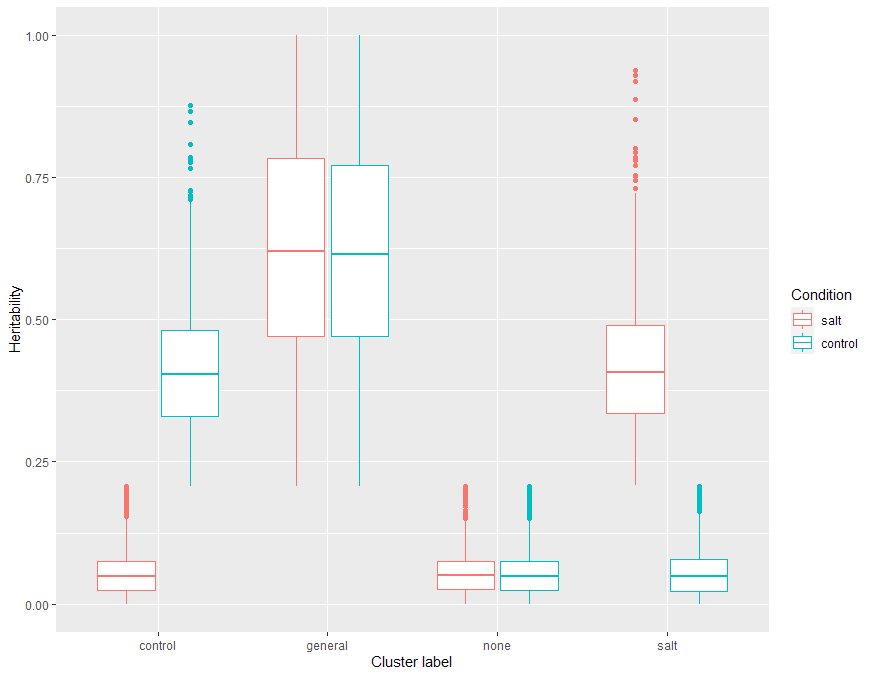
**

**Figure S3. Condition-specific Heritabilities.** Genes were organized into groups of significant heritability in salt treatment, control, both (general) or neither (none). Boxplots of GREML calculated heritability, *h*^2^, in each group is shown.

**
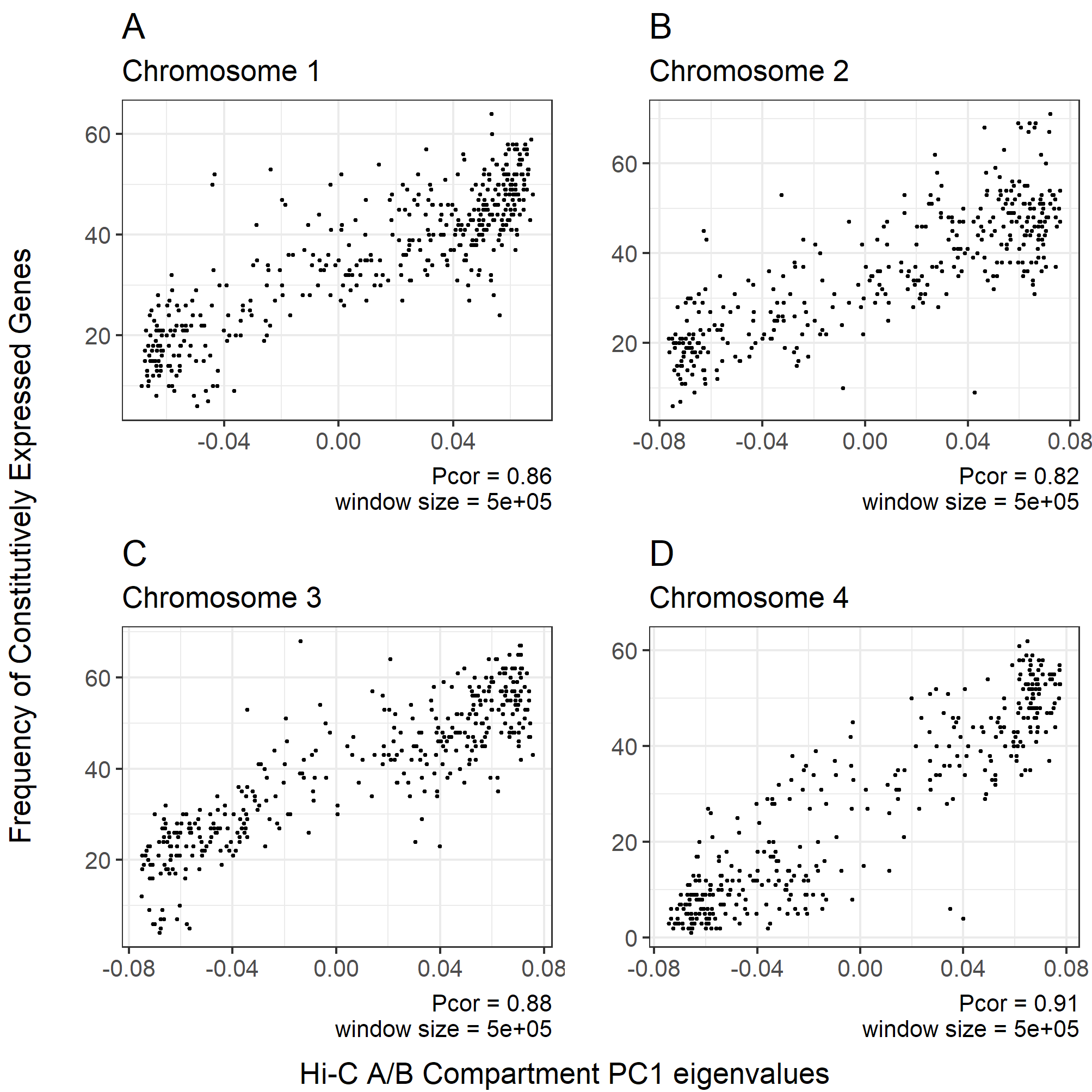
**

**Figure S4. Comparison of Hi-C and Gene Expression.** Plots A-D represent chromosomes 1, 2, 3, and 4 respectively. Eigenvectors of Hi-C A/B compartments are compared with the frequency of genes with constitutive expression (expressed in >95% of samples) using a fixed window size of 0.5 Mb.


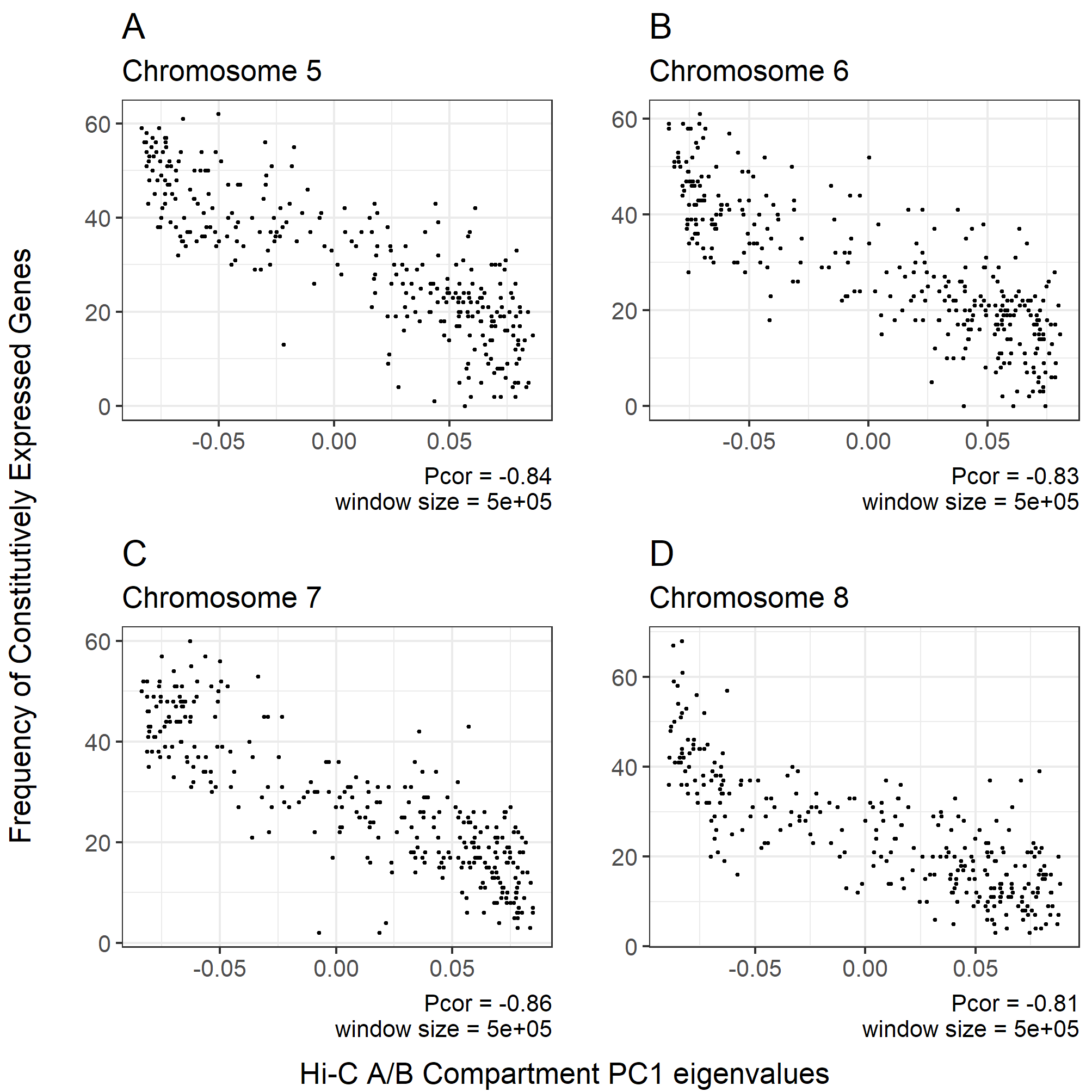


**Figure S5. Comparison of Hi-C and Gene Expression.** Plots A-D represent chromosomes 5, 6, 7, and 8 respectively. Eigenvectors of Hi-C A/B compartments are compared with the frequency of genes with constitutive expression (expressed in >95% of samples) using a fixed window size of 0.5 Mb.

**
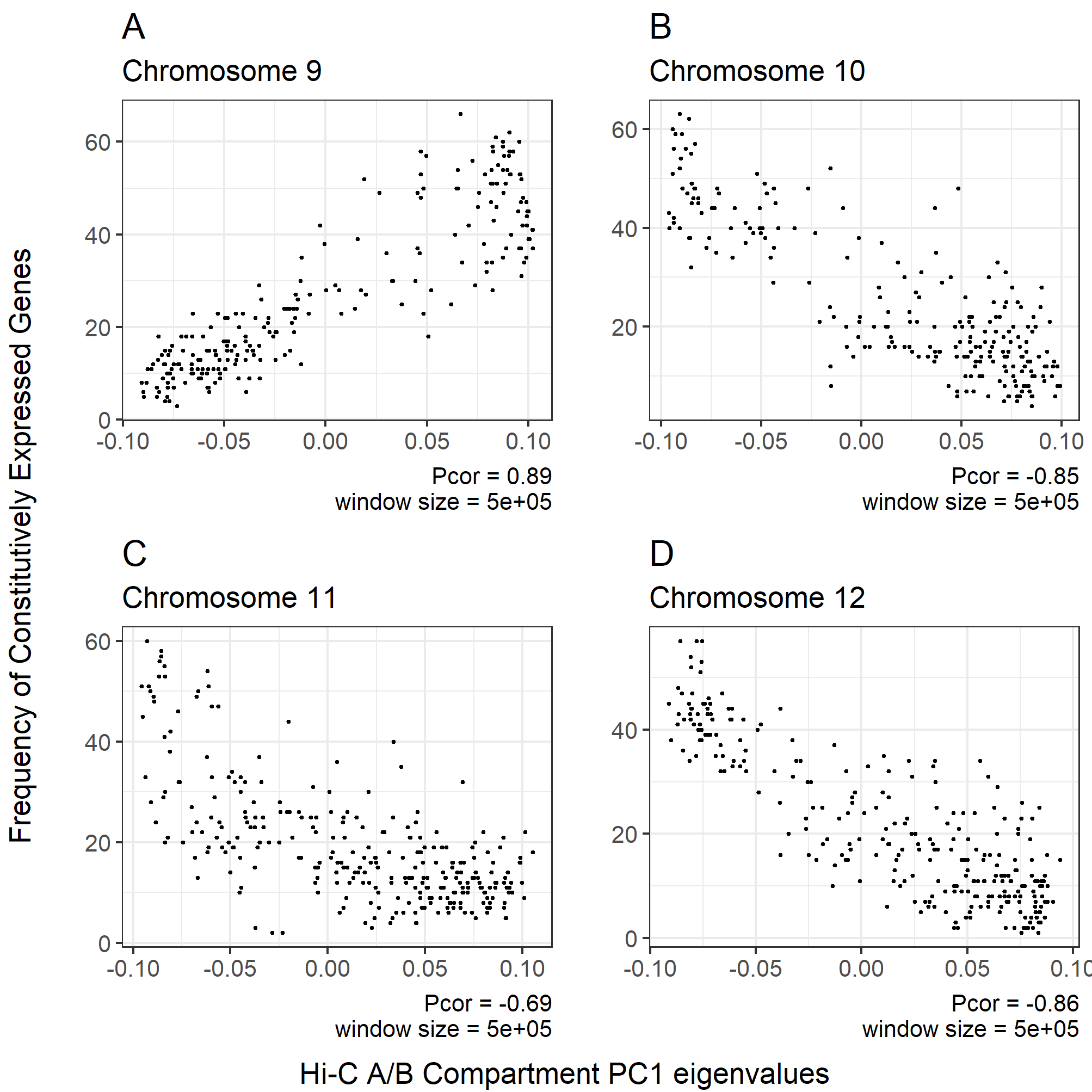
**

**Figure S6. Comparison of Hi-C and Gene Expression.** Plots A-D represent chromosomes 8, 10, 11, and 12 respectively. Eigenvectors of Hi-C A/B compartments are compared with the frequency of genes with constitutive expression (expressed in >95% of samples) using a fixed window size of 0.5 Mb.

**
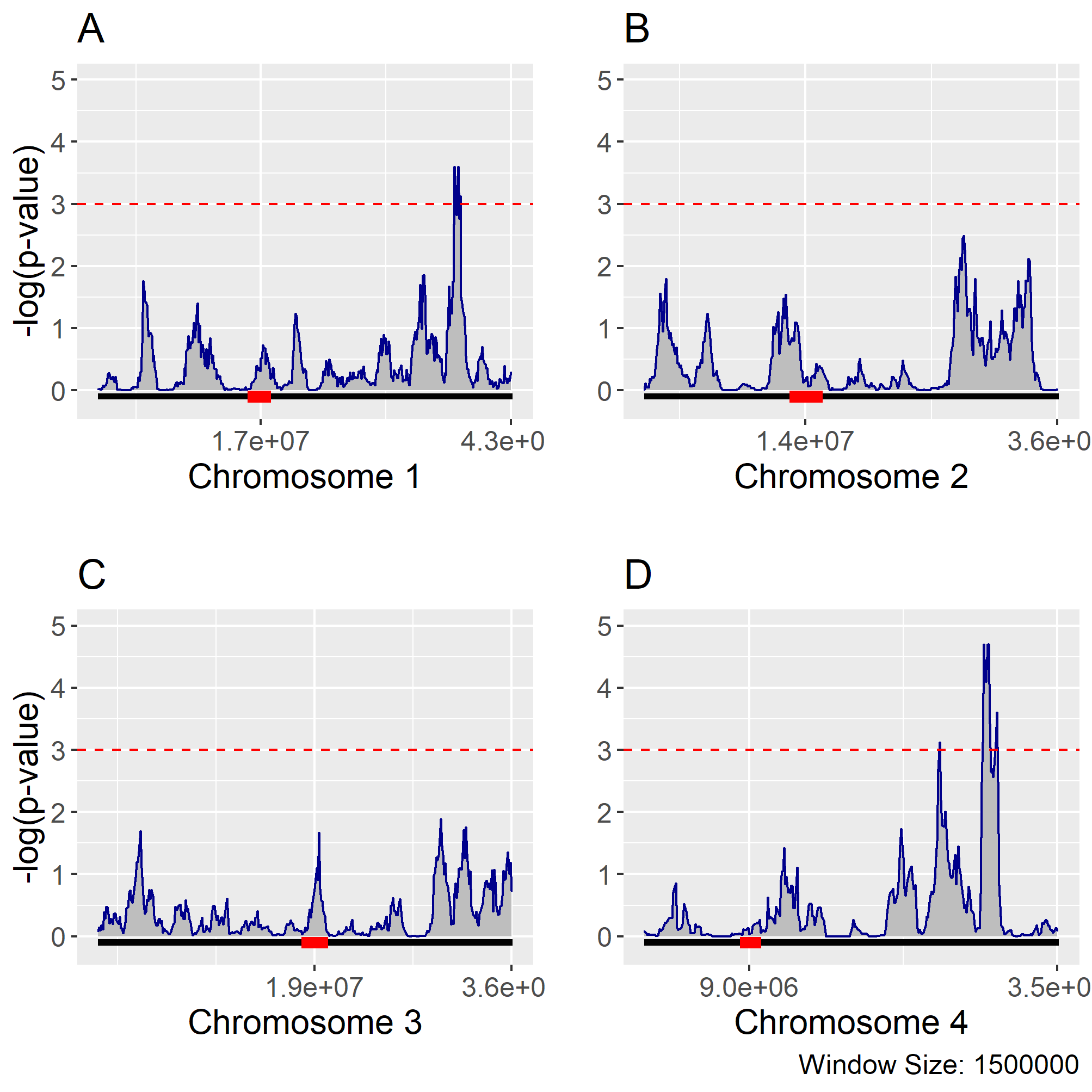
**

**Figure S7. Salt-specific Heritability Enrichment.** Plots A-D represent chromosomes 1, 2, 3, and 4 respectively. The black lines at the bottom of each plot represent the relative chromosome length, with the position and relative size of pericentromeric regions indicated by overlapping red boxes. Using a sliding window size of 1.5 Mb at 100 Kb intervals, chromosomes were tested for enrichment of genes with salt-specific heritability using all genes with heritable expression (salt-specific, optimal-specific, and general) as the null distribution. P-values were adjusted for multiple-testing using a permutation based approach. Using a critical value of 0.001, indicated by the dashed red line, significant windows enriched for salt-specific heritability were identified.

**
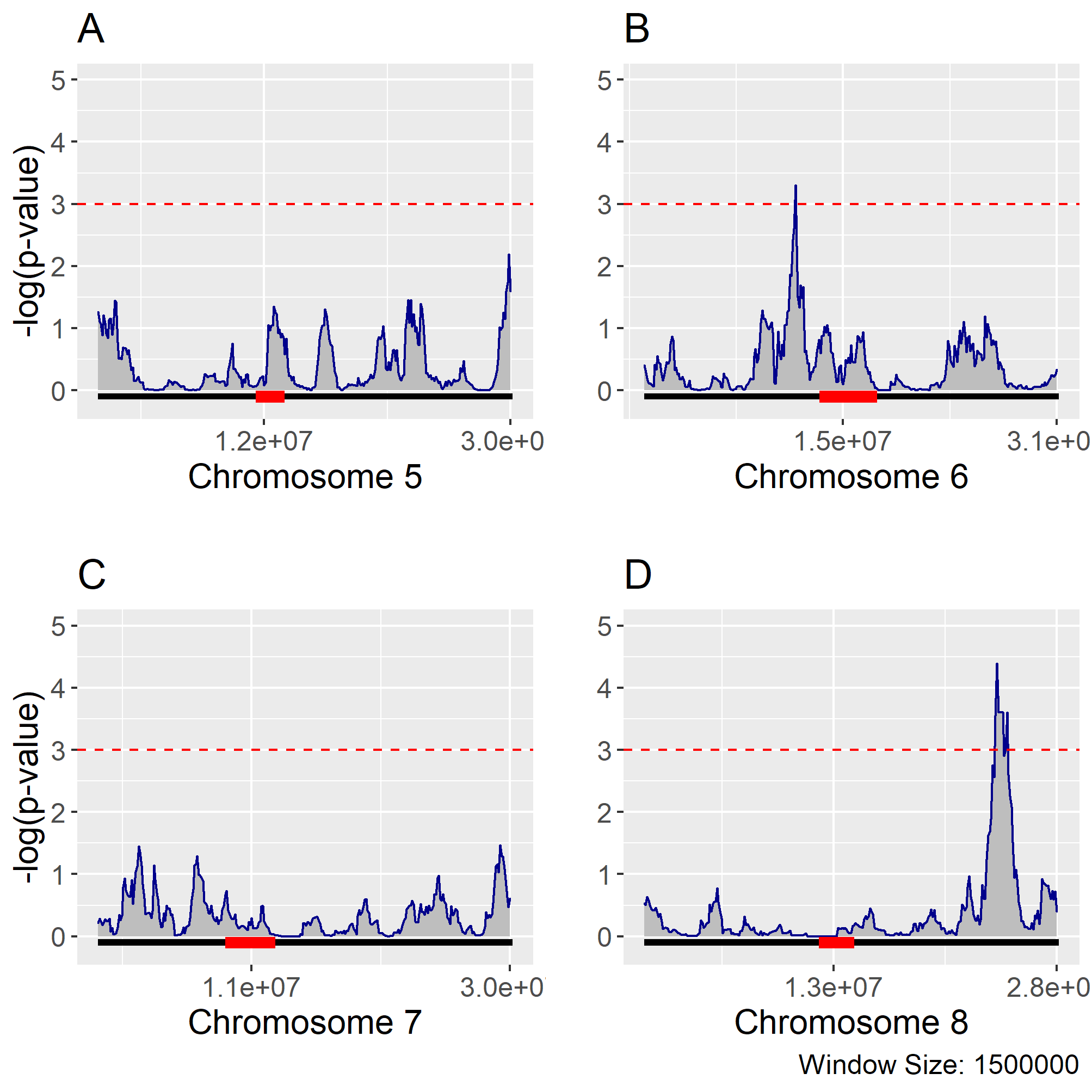
**

**Figure S8. Salt-specific Heritability Enrichment.** Plots A-D represent chromosomes 5, 6, 7, and 8 respectively. The black lines at the bottom of each plot represent the relative chromosome length, with the position and relative size of pericentromeric regions indicated by overlapping red boxes. Using a sliding window size of 1.5 Mb at 100 Kb intervals, chromosomes were tested for enrichment of genes with salt-specific heritability using all genes with heritable expression (salt-specific, optimal-specific, and general) as the null distribution. P-values were adjusted for multiple-testing using a permutation based approach. Using a critical value of 0.001, indicated by the dashed red line, significant windows enriched for salt-specific heritability were identified.

**
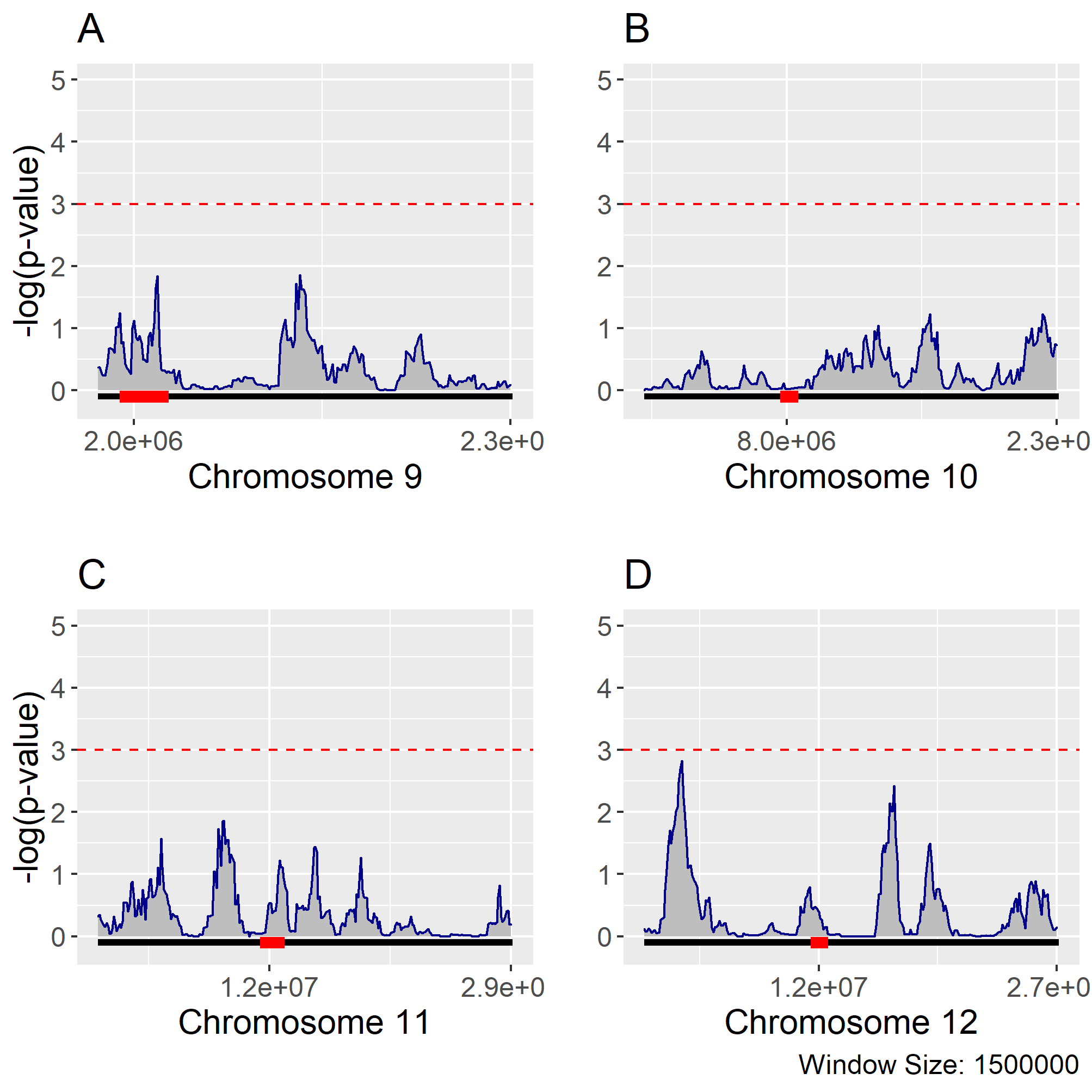
**

**Figure S9. Salt-specific Heritability Enrichment.** Plots A-D represent chromosomes 9, 10, 11, and 12 respectively. The black lines at the bottom of each plot represent the relative chromosome length, with the position and relative size of pericentromeric regions indicated by overlapping red boxes. Using a sliding window size of 1.5 Mb at 100 Kb intervals, chromosomes were tested for enrichment of genes with salt-specific heritability using all genes with heritable expression (salt-specific, optimal-specific, and general) as the null distribution. P-values were adjusted for multiple-testing using a permutation based approach. Using a critical value of 0.001, indicated by the dashed red line, significant windows enriched for salt-specific heritability were identified.


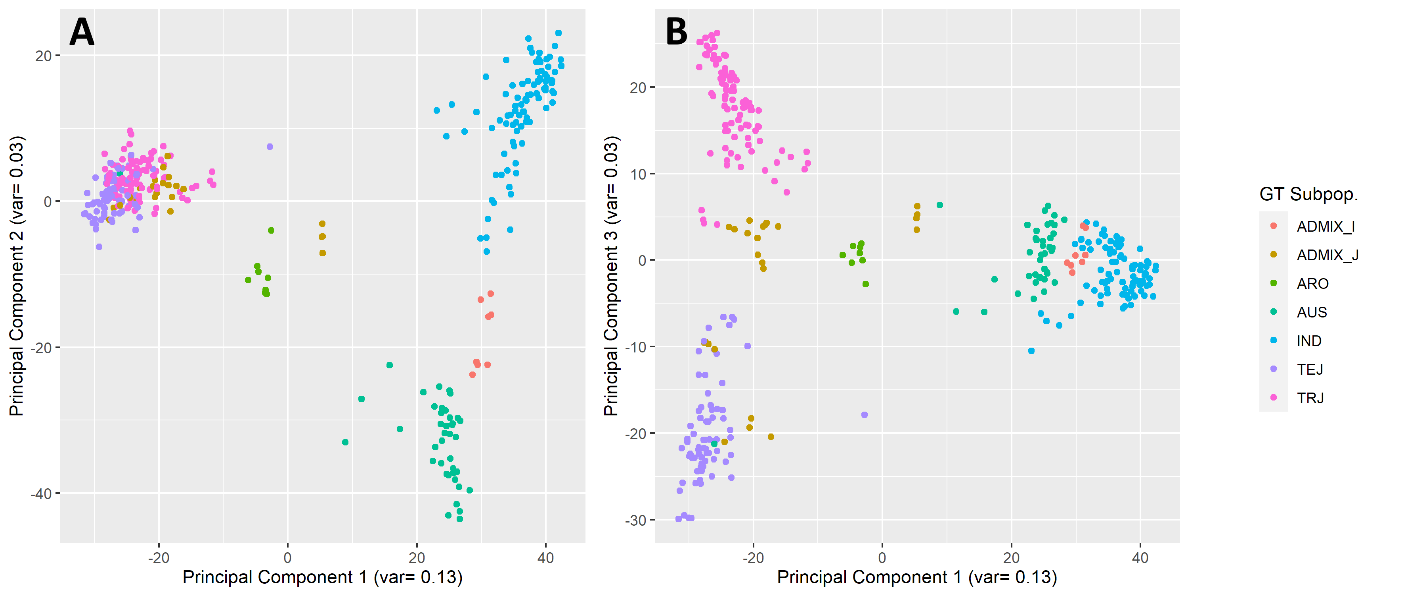


**Figure S10. PCA Using Ordinal Categorical Gene Expression.** Scatterplots of the top two principal components calculated from a gene expression matrix. RNA-seq samples were encoded as zero, one, or two if TPMs were zero, low, or high respectively. The top two components closely match principal components calculated using genotypes instead of gene expression (see Figure S11).


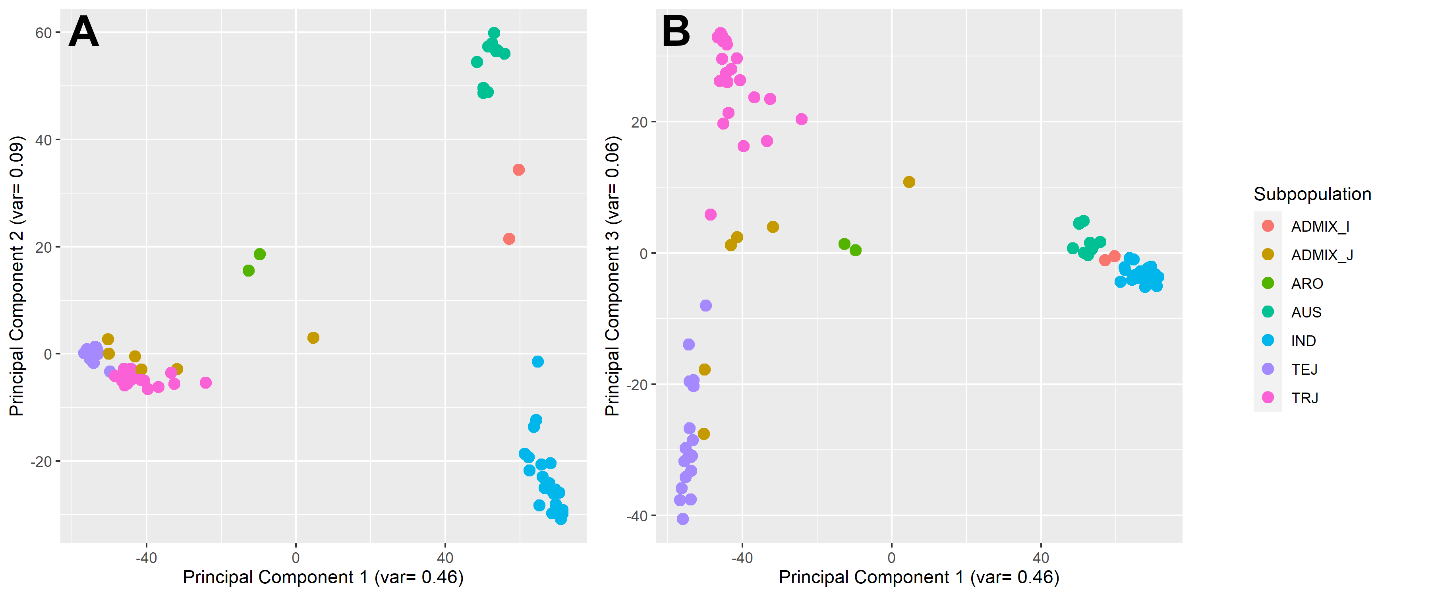


**Figure S11. Rice Diversity Panel 1 Population Structure.** Scatterplots of the top two principal components (PC) calculated from genotypes of the 84 individuals subset from the RDP1 panel. The sample distribution matches domestication history in rice. PC1 of both plots corresponds to variance between japonica (TEJ and TRJ) and indica (IND) varieties. PC2 of plot A captures variance between indica and aus varieties. PC2 of plot B captures variance between temperate (TEJ) and tropical (TRJ) japonica varieties. Hybrid varieties (ADMIX_I and ADMIX_J) and aromatic (ARO) varieties occupied areas between major subspecies clusters


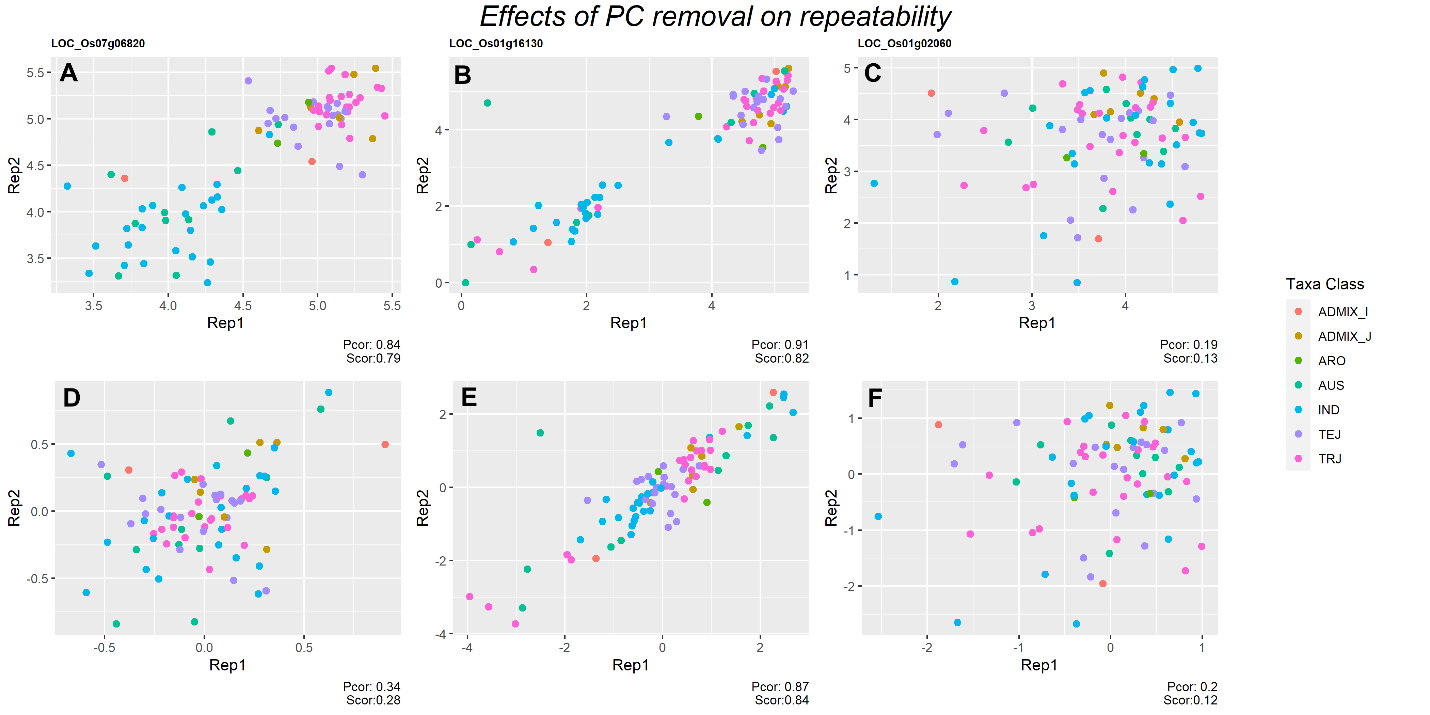


**Figure S12. Adjusting for population structure in Gene Expression.** Scatterplots A-C show unadjusted gene expression between replicate genotypes for three selected genes . Population structure can result in distinct clustering of samples.. Plots D-F show the same three genes after population structure is removed from gene expression. With correction for population structure, the gene in plot A would falsely indicate high correlation (i.e., repeatability). Plot D indicates no correlation implying no repeatability. The gene in plots B and E maintains correlation even when population structure is removed and the gene in plots C and F is largely unaffected.
